# Predictive value of high-resolution computed tomography radiomics for assessing the invasiveness of pulmonary adenocarcinoma presenting as ground-glass nodules: A retrospective study

**DOI:** 10.1097/MD.0000000000047964

**Published:** 2026-03-06

**Authors:** Wei Zhou, Jing Zhu, Yinshuo Mei, Yang Li, Ruonan Sun, Changjun Zheng

**Affiliations:** a Department of Radiology, Clinical Medical College, Chengdu Medical College, The First Affiliated Hospital of Chengdu Medical College, Chengdu, People’s Republic of China; b Department of Research and Development, Chengdu United Imaging Intelligence Co., Ltd., Chengdu, China; c Information Center, Clinical Medical College, Chengdu Medical College; The First Affiliated Hospital of Chengdu Medical College, Chengdu, People’s Republic of China.

**Keywords:** adenocarcinoma of the lung, ground-glass nodule, high-resolution computed tomography, radiomics

## Abstract

This study aims to explore the predictive value of radiomics based on high-resolution computed tomography for assessing the invasiveness of pulmonary adenocarcinoma manifested as ground-glass nodules (GGNs). We collected data from 360 GGNs cases confirmed as pulmonary adenocarcinoma by surgical pathology at the First Affiliated Hospital of Chengdu Medical College between January 2019 and December 2023. Clinical information and imaging features were also gathered. The cases were divided into a noninvasive group with 127 cases (35.3%) and an invasive lesion group with 233 cases (64.7%). A total of 279 GGN patients were randomly divided into a training group (195 cases) and a test group (84 cases), with a 7:3 ratio, and 81 cases were included in the validation group. Based on the selected clinical features and radiomics features of GGN, a radiomics model, a clinical model, and a combined clinical-radiomics model were constructed using LASSO and multivariate Logistic Regression. The predictive performance of the 3 models was evaluated and compared using the area under the curve, decision curve analysis, and DeLong test. In the validation set, the area under the curves for the radiomics model (using 29 radiomics features), the clinical model (mean length and air bronchogram), and the combined clinical-radiomics model were 0.856, 0.901, and 0.911, respectively. Decision curve analysis and DeLong’s test indicated that the combined clinical-radiomics model has higher value in predicting the invasiveness of GGNs than the other 2 models. The combined clinical-radiomics model demonstrates good performance in predicting the invasiveness of pulmonary adenocarcinoma presenting as GGNs. It has the potential to provide effective imaging methods for patients planning precise treatment.

## 1. Introduction

In recent years, low-dose high-resolution computed tomography (CT) has gained increasing attention in the diagnosis of ground-glass nodules (GGNs) in the lungs. Currently, many studies have reported imaging features that distinguish the benignity and malignancy of GGNs, but the features that demonstrate the extent of their pathological invasiveness remain unclear. In this study, we identified CT imaging features that indicate the extent of pathological invasiveness of GGNs.

GGNs are defined as areas of partially transparent and increased hazy density on CT imaging, without exhibiting obvious solid nodules.^[1]^ With the widespread use of low-dose high-resolution CT in recent years, it has garnered increasing attention in diagnosing GGNs in the lungs. Studies suggest that some GGNs may present manifestations of lung adenocarcinoma, especially pure ground-glass nodules (pGGNs), which may indicate precancerous or even cancerous lesions of the lungs.^[2]^ Most international guidelines suggest a conservative treatment approach for pGGNs. The percentage of malignancy was <1% for nodules smaller than 6 mm and 1% to 2% for nodules between 6 mm and 8 mm.^[2]^ These data suggest that some pGGNs are malignant and should not be overlooked. Features such as focal interstitial fibrosis, eosinophilic pneumonia, aspergillosis, bronchiolitis obliterans organizing pneumonia, and Wegener granulomatosis are favorable for diagnosing malignant lesions in pGGNs.^[3]^ However, it remains challenging to diagnose malignant lesions in pGGNs solely by visual assessment based on these CT imaging features, as they are influenced by scanning parameters, reconstruction algorithms, and observer experience.

Radiomics is a method that uses information extracted from CT images to indicate tumor heterogeneity and disease characteristics in a quantitative manner, and it plays an important role in individualized treatment, disease diagnosis, assessment of treatment efficacy, and prediction of prognosis.^[4,5]^ The main advantage of radiomics is that it provides quantitative information which goes beyond the visual assessment to reveal the underly biological features. Radiomics generally includes the following steps. First, the acquisition and preprocessing of medical images to ensure the quality and consistency of data. Second, the precise definition of regions of interest (ROIs). Third, the extraction of a large quantity of features from these interesting regions.^[6]^ Finally, these features are analyzed using statistical, machine learning, and deep learning methods to reveal patterns associated with disease diagnosis, treatment response, and disease prognosis. Currently, radiomics has received increasing attention in the diagnosis and evaluation of various diseases. Therefore, this study aims to establish a comprehensive model that is based on high-resolution CT radiomics to improve the diagnostic efficacy of pGGNs and provide information for clinical practice decision-making.

## 2. Materials and methods

### 2.1. Patient enrollment

This study retrospectively enrolled 360 patients with pathologically confirmed pulmonary adenocarcinoma presenting as GGNs at the First Affiliated Hospital of Chengdu Medical College between January 2019 and December 2023. The patient selection flowchart is illustrated in Figure S1, Supplemental Digital Content, https://links.lww.com/MD/R507. Inclusion criteria were: presence of a solitary GGN with a maximum diameter ≤ 3 cm; and availability of high-resolution thin-slice chest CT images (slice thickness ≤ 1 mm). Exclusion criteria included: diffuse lung disease, advanced lung cancer, or intrapulmonary metastases; and image quality or format unsuitable for radiomics analysis. Among the included cases, 279 scanned on a Siemens Definition AS+ 64-slice spiral CT scanner were randomly allocated to a training set (n = 195) and a test set (n = 84) in a 7:3 ratio. An additional 81 cases scanned on a United Imaging uCT530 40-slice spiral CT scanner were included as an independent validation set.

The collected data included: patient characteristics, such as age, gender, and smoking history; and CT morphological features of the lesions, including the mean length ((Maximum major diameter + Maximum minor diameter)/2, mm), mean CT attenuation (Hounsfield Units, HU), nodule type (mixed or pure GGN), location, and the presence of specific signs such as lobulation, pleural indentation, and air bronchogram.

### 2.2. Pathological diagnosis

Lung resection specimens were fixed in 4% formaldehyde immediately upon resection (within 30 minutes). Tissue sections were stained with hematoxylin and eosin (H&E) and subjected to immunohistochemical (IHC) analysis in the pathology department. Histopathological diagnoses were established by 2 experienced pathologists. In cases of diagnostic disagreement, a consensus was reached through joint review and discussion. Diagnostic criteria were based on the following classifications – atypical adenomatous hyperplasia (AAH): focal lesions (≤0.5 cm) exhibiting mild to moderate epithelial atypia, growing along alveolar or respiratory bronchiolar walls, without interstitial inflammation or fibrosis. Adenocarcinoma in situ (AIS): localized lesions (≤3.0 cm) with lepidic-pattern epithelial growth confined to alveolar structures, showing no stromal, vascular, or pleural invasion. Minimally invasive adenocarcinoma (MIA): small focal lesions (≤3.0 cm) primarily exhibiting lepidic growth, with an invasive component measuring ≤ 0.5 cm in greatest dimension. Invasive adenocarcinoma (IAC): lesions (≤3.0 cm) featuring an invasive focus > 0.5 cm. Based on these criteria, AAH and AIS were categorized as the noninvasive group (127 cases), while MIA and IAC were classified as the invasive group (233 cases).

### 2.3. CT examination

The Siemens Definition AS + 64-slice spiral CT scanner and the United Imaging uCT530 40-slice spiral CT scanner were used for patient examination. Patients were scanned in the supine position with arms raised and head first, holding their breath at the end of inhalation. The scanning range was from the lung apex to the lung base. All parameters were obtained from non-contrast CT scans, with images analyzed using lung window settings (window width 1600 HU, window level −600 HU). The scanning parameters were as follows: Siemens Definition AS+ 64-slice spiral CT: tube voltage 120 kV; tube current: automatic tube current modulation; pitch: 1.2; slice thickness: 5.0 mm; image matrix: 512 × 512; field of view: 326 mm. United Imaging uCT530 40-slice spiral CT: tube voltage 120 kV; tube current: automatic tube current modulation; pitch: 0.8688; slice thickness: 5.0 mm; image matrix: 512 × 512; field of view: 350 mm. Images were reconstructed using standard algorithms to produce 1 mm-thick axial images.

### 2.4. CT image processing

All the initial thin-layer lung window CT images of the patients were gathered and imported into the uAI research portal (version 20230915). An experienced radiologist A (Wei Zhou) identified the nodules without prior knowledge of the pathological diagnosis and manually delineated the margins of the nodules layer by layer. The platform software automatically generated the regions of interest (ROIs). To guarantee data accuracy, the shape and margins of the nodules were meticulously reviewed during the delineation process to prevent confusion with vessels, bronchi, and vacuolar components. One month later, radiologists A and B (Yinshuo Mei) re-drew the regions of interest (ROIs) to calculate the intraclass correlation coefficient (ICC) used to assess the consistency and repeatability of the extracted features. Features with an ICC > 0.8 were included in subsequent analyses.

### 2.5. Pathological diagnosis

Surgical lung specimens were fixed with 4% formaldehyde within half an hour after surgery. The specimen sections were stained with hematoxylin-eosin (H&E staining) and subjected to immunohistochemistry at the pathology department. Two pathologic specialists collaborated to perform the diagnosis. When there were discrepancies in diagnoses, consensus was reached through general discussion among pathologic specialists. The diagnostic criteria were as follows: AAH: lesions (≤0.5 cm) with mild to moderate epithelial atypia growing along the alveolar wall or respiratory bronchial wall, and no interstitial inflammatory reaction and fibrosis; AIS: focal lesion (≤3.0 cm) epithelial growth along the alveolar wall without interstitial, vascular, or pleural infiltration; MIA: focal lesion (≤3.0 cm), alveolar cells grow mainly in an adnexal pattern with infiltrating foci ≤ 3.0 cm. IAC: focal lesion (≤3.0 cm) with infiltrating foci > 0.5 cm.

### 2.6. Radiomics feature extraction and selection

To ensure reproducibility, this study followed the Imaging Biomarker Standardization Initiative (IBSI) guidelines for image preprocessing and feature extraction. All chest CT images (lung window) were resampled to an isotropic voxel size of 1.0 × 1.0 × 1.0 mm^3^ using B-spline interpolation to reduce spatial resolution anisotropy. Gray-scale intensities were discretized with a fixed bin width of 25, and intensity normalization was applied to minimize variations from scanning protocols and equipment, improving feature robustness and comparability. A total of 2264 radiomics features were extracted from each region of interest (ROI). These features comprised: first-order statistics; shape-based features (n = 14); gray-level co-occurrence matrix (GLCM, n = 21); gray-level run-length matrix (GLRLM, n = 16); gray-level size zone matrix (GLSZM, n = 16); neighboring gray-tone difference matrix (NGTDM, n = 5); and gray-level dependence matrix (GLDM, n = 14). Additionally, features were derived after applying the following filter transforms: Mean (Boxmean), Additive Gaussian Noise, Binomial Blur, Curvature Flow, Sigma Image (Boxsigmaimage), Laplacian of Gaussian (LoG) at sigma values 0.5, 1.0, 2.0, and 4.0 mm, Wavelet, Laplacian Sharpening, Discrete Gaussian, Mean, Speckle Noise, Recursive Gaussian, and Shot Noise.

To ensure the comparability of radiomic features and enhance model robustness and generalizability, *Z*-score normalization was applied to each feature. This was performed using the mean and standard deviation calculated exclusively from the training set, which were then applied to the training, test and validation sets to prevent data leakage. Subsequently, feature stability screening was conducted on the initial feature set, retaining only features with an ICC > 0.80. Following this, feature selection was performed using the Least Absolute Shrinkage and Selection Operator (LASSO) algorithm. This method introduces an L1 regularization penalty term to shrink the coefficients of less informative features towards 0, thereby achieving sparse modeling. The optimal regularization strength parameter (λ) was determined via 10-fold cross-validation,leading to the selection of 29 radiomic features with the highest predictive power for subsequent model construction. All analyses were implemented in Python 3.9.7 using the scikit-learn 1.0.2 library on a workstation running Ubuntu 20.04 LTS, equipped with 64 GB of RAM and an Intel Core i9 processor.

### 2.7. Model development and evaluation

To identify independent predictors for the construction of the clinical model, we initially performed univariate analysis on all baseline clinical characteristics (such as age, gender, smoking history, location of nodules, lobular signs, and vacuolar signs, etc). Variables exhibiting statistical significance (*P* < .05) were subsequently subjected to variance inflation factor (VIF) tests to evaluate multicollinearity. Only features with a VIF value less than 5 were incorporated into the multivariate Logistic Regression (LR) analysis. Ultimately, the features that maintained statistical significance (*P* < .05) in the multivariate analysis were utilized to develop the clinical prediction model. Eventually, 3 models were constructed: a clinical model based on clinical features, a radiomics model, and a combined clinical-radiomics model.

Three machine learning algorithms, including LR, Random Forest (RF), and Support Vector Machine (SVM), were employed to construct predictive models. Following model training, their performance was rigorously evaluated using receiver operating characteristic (ROC) curve analysis. The area under the ROC curve (AUC) along with its 95% confidence interval (95% CI) was calculated to assess the overall discriminative ability of each model. Additionally, a comprehensive set of metrics including sensitivity, specificity, accuracy, precision, and F1-score were computed to provide a detailed assessment of classification performance across different aspects.

To evaluate the agreement between predicted probabilities and actual observed outcomes, the calibration of each model was visually examined using calibration curves. Furthermore, decision curve analysis was performed to estimate the clinical utility of the models. This analysis quantified the net benefit derived from each model across a range of threshold probabilities, facilitating the assessment of their potential value in supporting clinical decision-making for interventions based on risk stratification.

### 2.8. Statistical analysis

This statistical analysis was performed with SPSS 25.0 (Chengdu, China). Categorical variables are expressed as count with percentage, and continuous variables are presented as means with standard deviations (SD). Variables were analyzed using chi-square test or the Fisher exact test for the categorical variables and the Mann–Whitney *U* test for the continuous variables as appropriate, respectively. A 2-sided *P* < .05 was considered to represent statistically significant.

## 3. Results

### 3.1. Patient demographic characteristics

A total of 360 patients with GGNs were included in this study, of whom 183 (50.8%) were pure GGNs (pGGN) and 177 (49.2%) were mixed GGNs (mGGN). The noninvasive group (AAH/AIS) consisted of 127 (35.3%) cases, while the invasive group (MIA/IAC) consisted of 233 (64.7%) cases. The clinical characteristics were shown in Table 1, and the CT indications were shown in Table 2. These nodules were located at the superior lobe of right lung included 164 (45.6%) cases, the middle lobe of right lung included 29 (8.1%) cases, the inferior lobe of right lung included 44 (12.2%) cases, the superior lobe of left lung included 84 (23.3%) cases, and the inferior lobe of left lung included 39 (10.8%) cases. The 279 patients with GGNs were randomly divided into a training group of 195 cases and a testing group of 84 cases, with a 7:3 ratio, and an additional 81 cases were included in the validation group (Table 1). Among the training, testing, and validation groups, there were no significant differences between the noninvasive group and the invasive group in terms of age (*P* = .4816), sex (*P* = .5019), history of smoking (*P* = .0384), nodule classification (*P* = .0519), nodule position (*P* = .7761), vacuole sign (*P* = .2284), or pleura involve (*P* = .0013). However, significant differences were observed among the 3 groups in the mean length (*P* < .0001), lobulation sign (*P* < .0001), burr (*P* = .0002), and air bronchogram (*P* = .0006), and the results were presented in Table 1.

**Table 1:** Analysis of clinical and CT characteristics between noninvasive and invasive groups.

Patient characteristics	Training cohort		*P* value	Testing cohort		*P* value	Validation cohort		*P* value
	Noninvasive group (n = 67)	Invasive group (n = 128)		Noninvasive group (n = 29)	Invasive group (n = 55)		Noninvasive group (n = 31)	Invasive group (n = 50)	
Age (yr)	52.57 ± 11.45	60.56 (±10.42)	<.0001	51.28 ± 11.50	60.91 ± 10.01	.0003	54.26 ± 12.25	56.42 ± 11.44	.4816
Sex			.8822			.7978			.5019
Female	48 (71.6%)	93 (72.7%)		22 (75.9%)	39 (70.9%)		18 (58.1%)	25 (50.0%)	
Male	19 (28.4%)	35 (27.3%)		7 (24.1%)	16 (29.1%)		13 (41.9%)	25 (50.0%)	
Mean length (mm)	7.82 ± 2.26	15.4 ± 5.86	<.0001	7.62 ± 1.94	14.45 ± 5.49	<.0001	7.806 ± 2.344	15.58 ± 6.792	<.0001
Mean CT (Hu)	−622.0 ± 112.1	−544.4 ± 129.1	<.0001	−592.4 ± 109.6	−540.5 ± 140.9	.1462	−656.7 ± 104.2	−644.0 ± 78.61	.3455
Nodule classification			<.0001			.023			.0519
mGGO	18 (26.9%)	87 (68.0%)		10 (34.5%)	35 (63.6%)		6 (19.4%)	21 (42.0%)	
pGGO	49 (73.1%)	41 (32.0%)		19 (65.5%)	20 (37.4%)		25 (80.6%)	29 (58.0%)	
Nodule position			.6956			.6973			.7761
Superior lobe of right lung	31 (46.3%)	62 (48.4%)		9 (31.0%)	25 (45.5%)		15 (48.4%)	22 (44.0%)	
Middle lobe of right lung	6 (9.0%)	9 (7.0%)		5 (17.2%)	4 (7.3%)		1 (3.2%)	4 (8.0%)	
Inferior lobe of right lung	8 (11.9%)	20 (15.6%)		7 (24.2%)	5 (9.1%)		2 (6.4%)	2 (4.0%)	
Superior lobe of left lung	13 (19.4%)	24 (18.8%)		6 (20.7%)	19 (34.5%)		10 (32.3%)	12 (24.0%)	
Inferior lobe of left lung	9 (13.4%)	13 (10.2%)		2 (6.9%)	2 (3.6%)		3 (9.7%)	10 (20.0%)	
Lobulation			<.0001			<.0001			<.0001
Yes	21 (31.3%)	91 (71.1%)		7 (24.1%)	42 (76.4%)		17 (54.8%)	47 (94%)	
No	46 (68.7%)	37 (28.9%)		22 (75.9%)	13 (23.6%)		14 (45.2%)	3 (6%)	
Pleura involve			<.0001			.0547			.0013
Yes	13 (19.4%)	66 (51.6%)		6 (20.7%)	24 (43.6%)		6 (19.4%)	28 (56%)	
No	54 (80.6%)	62 (48.4%)		23 (79.3%)	31 (56.4%)		25 (80.6%)	22 (44%)	
Burr			<.0001			.0086			.0002
Yes	14 (23.7%)	71 (52.9%)		5 (36.4%)	26 (45.1%)		5 (11.8%)	29 (63.8%)	
No	53 (76.3%)	57 (47.1%)		24 (63.6%)	27 (54.9%)		26 (88.2%)	21 (36.2%)	
Air bronchogram			<.0001			.0153			.0006
Yes	28 (41.8%)	93 (72.3%)		15 (51.7%)	43 (78.2%)		7 (22.6%)	31 (62%)	
No	39 (58.2%)	35 (27.3%)		14 (48.3%)	12 (21.8%)		24 (77.4%)	19 (38%)	
Vacuole			.1012			.0487			.2284
Yes	15 (19.7%)	44 (35.3%)		5 (27.3%)	22 (39.2%)		3 (5.9%)	11 (25.5%)	
No	52 (80.3%)	84 (64.7%)		24 (72.7%)	32 (60.8%)		29 (94.1%)	39 (74.5%)	
History of smoking			.5101			.5308			.0384
Yes	7 (10.5%)	18 (14.3%)		3 (12.1%)	9 (15.7%)		2 (8.8%)	13 (25.5%)	
No	60 (89.5%)	110 (85.7%)		26 (87.9%)	46 (84.3%)		29 (91.2%)	37 (74.5%)	

Statistical analysis was performed by SPSS 25.0. Data that conformed to a normal distribution were expressed as mean ± standard deviation, and comparisons between groups were made using the independent samples *t*-test. Categorical variables were expressed as the number of cases and percentages, and comparisons between groups were performed using the chi-square test or Fisher’s exact test. All data with a *P* < .05 were considered statistically significant.

CT = computed tomography.

**Table 2:** Results of univariate and multivariate Logistic Regression analysis.

Variables	Univariate analysis			Multivariate analysis		
	Exp (B)	95% CI	*P*	Exp (B)	95% CI	*P*
Age	1.045	1.017–1.073	.001	1.015	0.984–1.048	.346
Mean long-to-short axis ratio	1.220	1.132–1.315	.000	1.134	1.032–1.247	.009
Mean CT value	1.005	1.002–1.008	.000	1.002	0.999–1.005	.175
Solid component	4.434	2.398–8.197	.000	0.567	0.254–1.264	.166
Lobulation sign	5.384	2.879–10.069	.000	0.517	0.244–1.093	.084
Pleural indentation sign	2.203	1.206–4.025	.010	1.644	0.700–3.860	.254
Spicule sign	3.625	1.912–6.873	.000	0.701	0.304–1.619	.406
Air bronchogram	3.834	2.070–7.099	.000	0.432	0.208–0.897	.024
Vacuole sign	2.218	1.125–4.372	.021	0.440	0.191–1.013	.054

The statistical analysis was performed with SPSS 25.0. All data with a *P* < .05 were considered statistically significant.

CT = computed tomography.

Before including the significant clinical features (*P* < .05) from the univariate analysis of the training group in the multivariate LR analysis, we evaluated the multicollinearity among the variables. By calculating the VIF, it was confirmed that the VIF of all included variables was <2 (the highest value was 1.71). The results are detailed in Table S2, Supplemental Digital Content, https://links.lww.com/MD/R507, indicating that there were no serious collinearity issues. The results of the multivariate LR analysis are shown in Table 2. As a result, the mean length and air bronchogram of GGNs were identified as independent factors for diagnosing invasive pulmonary adenocarcinoma (*P* < .05). The cutoff value for the mean length was calculated to be 0.85 cm based on the Youden index (OR = 1.134, *P* = .009), and with the AUC, sensitivity, and specificity of 0.765, 0.815, and 0.579, respectively.

### 3.2. Model development and validation

**Figure 1.**
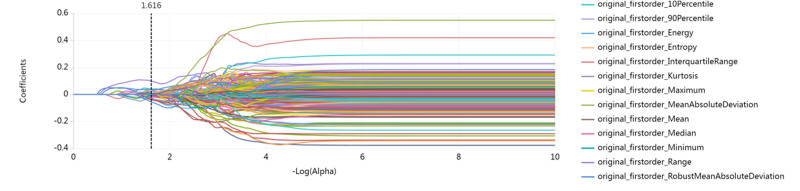
Feature selection of radiomics features using the LASSO model. LASSO = Least Absolute Shrinkage and Selection Operator.

**Figure 2.**
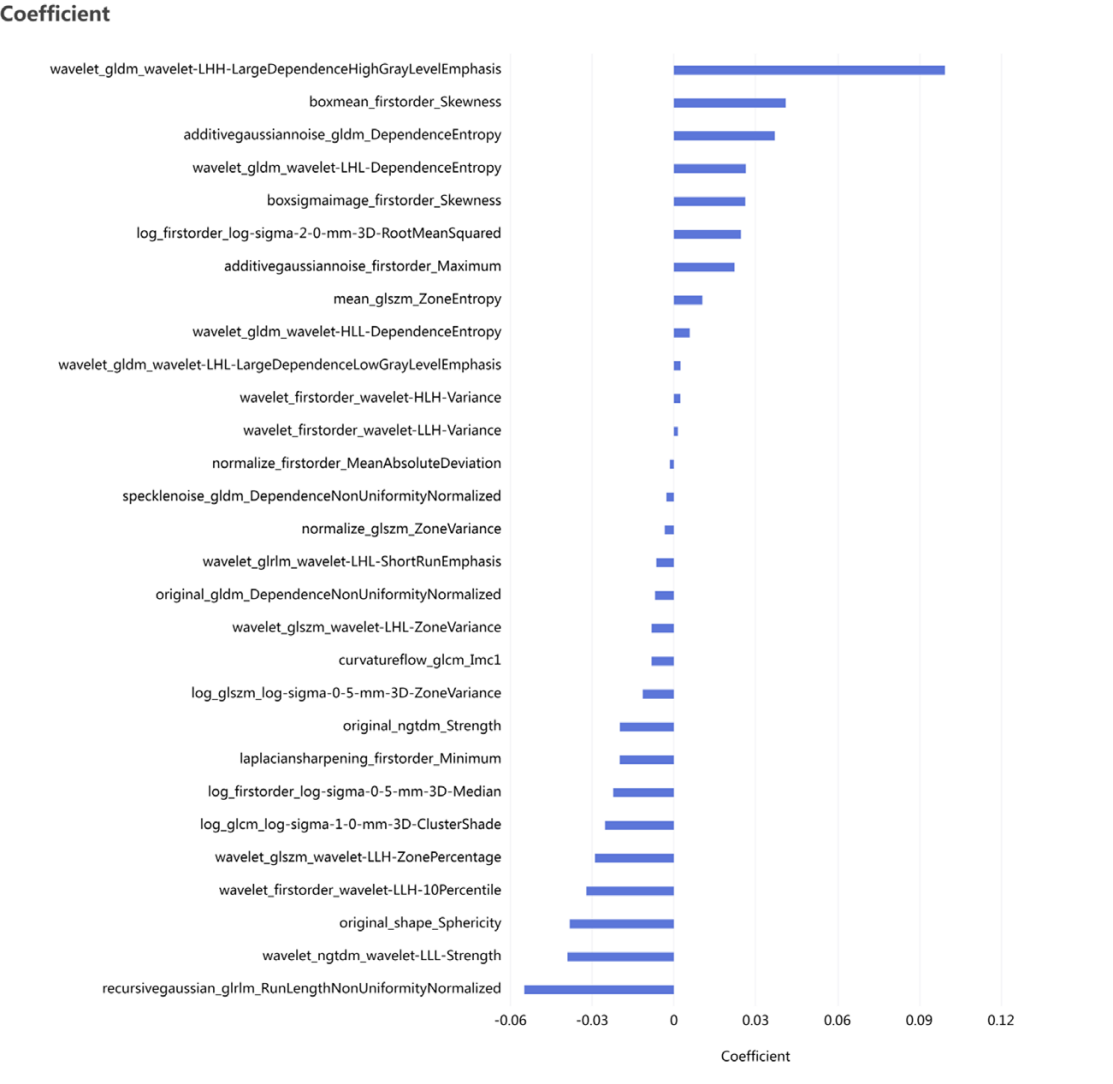
The 29 feature parameters and their corresponding coefficients retained after LASSO dimensionality reduction. LASSO = Least Absolute Shrinkage and Selection Operator.

The LASSO path was shown in Figure 1, and 29 specific features were ultimately selected, as shown in Figure 2. Through LASSO regression with *k*-fold (*k* = 10) cross-validation, the optimal λ value was determined to be 0.0242.

The 3 models (radiomics model, clinical model, and combined model) were evaluated using LR, RF, and SVM algorithms. The AUC (95% CI), sensitivity, specificity, accuracy , precision, and F1 score for each model are presented in Table 3. In the test set, the Combined Model built with LR yielded the highest AUC (0.923). Pairwise DeLong’s tests on the test set ROC curves for the Combined Model indicated no statistically significant difference between LR and RF (*P* = .353) or between LR and SVM (*P* = .802; see Table S1, Supplemental Digital Content, https://links.lww.com/MD/R507). Considering this statistical equivalence in performance, alongside the enhanced clinical interpretability and lower computational complexity of LR, we selected the LR-based models for final presentation and clinical discussion. Therefore, the radiomics model LR, clinical model LR, and combined clinical-radiomics model LR are hereafter reported. The AUCs in the validation set for these 3 models were 0.856, 0.901, and 0.911, respectively, as detailed in Table 4.

**Table 3:** Diagnostic performance of the radiomics model, clinical model, and combined model using Logistic Regression, Random Forest, and Support Vector Machine algorithms in the training and testing sets.

Model	Group	Classifier	AUC (95% CI)	Sensitivity	Specificity	ACC	Precision	F1Score
Radiomics	Train	Logistic Regression	0.988 (0.976–0.999)	0.805	0.970	0.862	0.981	0.884
	Test		0.918 (0.861–0.974)	0.818	0.862	0.833	0.918	0.865
	Train	Random Forest	0.975 (0.959–0.992)	0.844	0.955	0.882	0.973	0.904
	Test		0.902 (0.839–0.964)	0.836	0.828	0.833	0.902	0.868
	Train	Support Vector Machine	0.987 (0.975–1.000)	0.672	1.000	0.785	1.000	0.804
	Test		0.902 (0.839–0.966)	0.691	0.966	0.786	0.974	0.809
Clinical	Train	Logistic Regression	0.929 (0.894–0.963)	0.953	0.612	0.836	0.824	0.884
	Test		0.923 (0.868–0.978)	0.964	0.724	0.881	0.869	0.914
	Train	Random Forest	0.932 (0.899–0.966)	0.773	0.910	0.821	0.943	0.850
	Test		0.904 (0.837–0.972)	0.782	0.862	0.810	0.915	0.843
	Train	Support Vector Machine	0.923 (0.888–0.959)	0.797	0.910	0.836	0.944	0.864
	Test		0.918 (0.861–0.976)	0.800	0.897	0.833	0.936	0.863
Combine	Train	Logistic Regression	0.985 (0.973–0.997)	0.930	0.910	0.923	0.952	0.941
	Test		0.923 (0.869–0.978)	0.945	0.724	0.869	0.867	0.904
	Train	Random Forest	0.984 (0.971–0.998)	0.953	0.925	0.944	0.961	0.957
	Test		0.909 (0.847–0.971)	0.927	0.724	0.857	0.864	0.895
	Train	Support Vector Machine	0.985 (0.974–0.997)	0.930	0.881	0.913	0.937	0.933
	Test		0.922 (0.868–0.977)	0.927	0.690	0.845	0.850	0.887

The characteristic curve (ROC) was plotted to calculate the area under the curve (AUC) and analyze the sensitivity and specificity. All data with a *P* < .05 were considered statistically significant.

ACC = accuracy, AUC = area under the curve, CI = confidence interval, ROC = receiver operating characteristic.

**Table 4:** Diagnostic performance of the radiomics model, clinical model, and combined model in the validation set.

Model	Group	AUC	Sensitivity	Specificity	Accuracy	Precision	f1_score
Radiomics	Validation	0.856 (0.768–0.944)	0.8	0.806	0.802	0.87	0.833
Clinical	Validation	0.901 (0.83–0.972)	0.54	0.968	0.704	0.964	0.692
Combine	Validation	0.911 (0.844–0.977)	0.92	0.742	0.852	0.852	0.885

The characteristic curve (ROC) was plotted to calculate the area under the curve (AUC) and analyze the sensitivity and specificity. All data with a *P* < .05 were considered statistically significant.

AUC = area under the curve, ROC = receiver operating characteristic.

### 3.3. Performance of the clinical imaging model, radiomics model, and combined model

**Figure 3.**
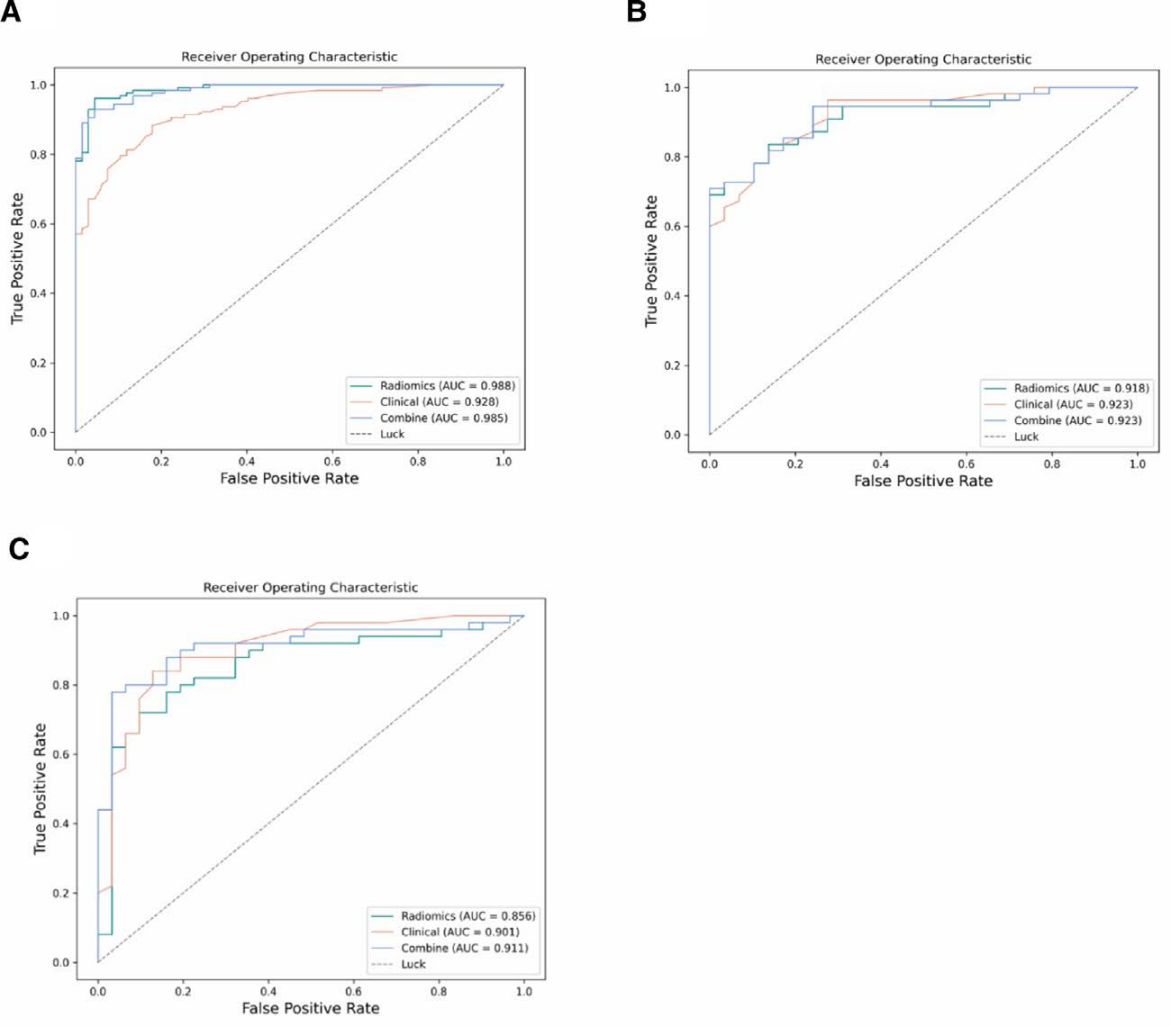
ROC curves of the 3 models in the training set (A) testing set (B), and validation set (C). AUC = area under the curve, ROC = receiver operating characteristic.

The ROC curves and AUC values for the radiomics model LR, clinical model LR, and combined clinical-radiomics model LR in the training, testing, and validation sets are shown in Figure 3. Notably, the radiomics model LR and the combined model LR performed similarly in the training set, with AUCs of 0.988 and 0.985, and sensitivities of 0.805 and 0.93 (Fig. 3A), respectively. In the testing set, both the clinical model LR and the combined model LR achieved AUCs of 0.923, with sensitivities of 0.964 and 0.945, respectively (Fig. 3B). The combined model LR demonstrated the best performance in the validation set, with an AUC of 0.911 and a sensitivity of 0.92 (Fig. 3C).

**Figure 4.**
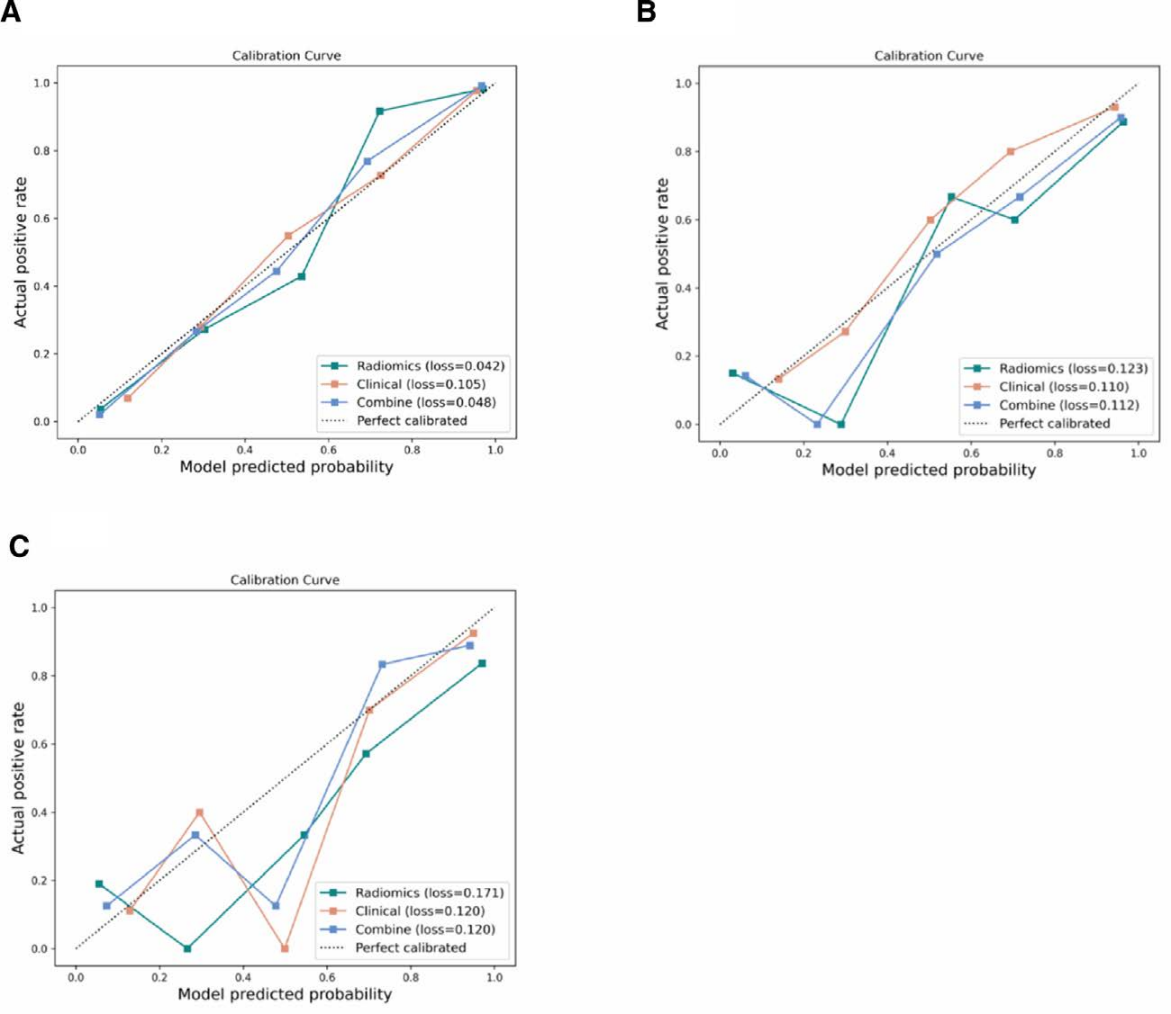
Calibration curves of the 3 models predicting GGNs invasiveness in the training set (A), testing set (B), and validation set (C). The *x*-axis represents the predicted probability, and the *y*-axis represents the actual probability. The diagonal dashed line labeled “Perfectly calibrated” represents the ideal situation where the predicted probability is equal to the actual probability. The closer the calibration curve is to the diagonal line, the better the predictive performance. GGN = ground-glass nodule.

The calibration performance of the radiomics, clinical, and combined models for predicting GGN invasiveness was assessed using calibration curves across the training, testing, and validation sets, as shown in Figure 4. In the training set (Fig. 4A), the combined model (Ioss = 0.048) and the radiomics model (Ioss = 0.042) demonstrated excellent calibration, with curves closely approximating the ideal line. The clinical model (Ioss = 0.105) showed good but slightly lower calibration. In the testing set (Fig. 4B), all 3 models maintained good calibration, with Ioss values of 0.123 (radiomics), 0.110 (clinical), and 0.112 (combined), indicating consistent generalization performance. In the external validation set (Fig. 4C), the combined and clinical models both achieved identical and favorable calibration (Ioss = 0.120), whereas the radiomics model exhibited moderate deviation (Ioss = 0.171). Overall, the combined model consistently showed well-calibrated predictions across all cohorts, supporting its reliability for clinical probability estimation of GGN invasiveness.

**Figure 5.**
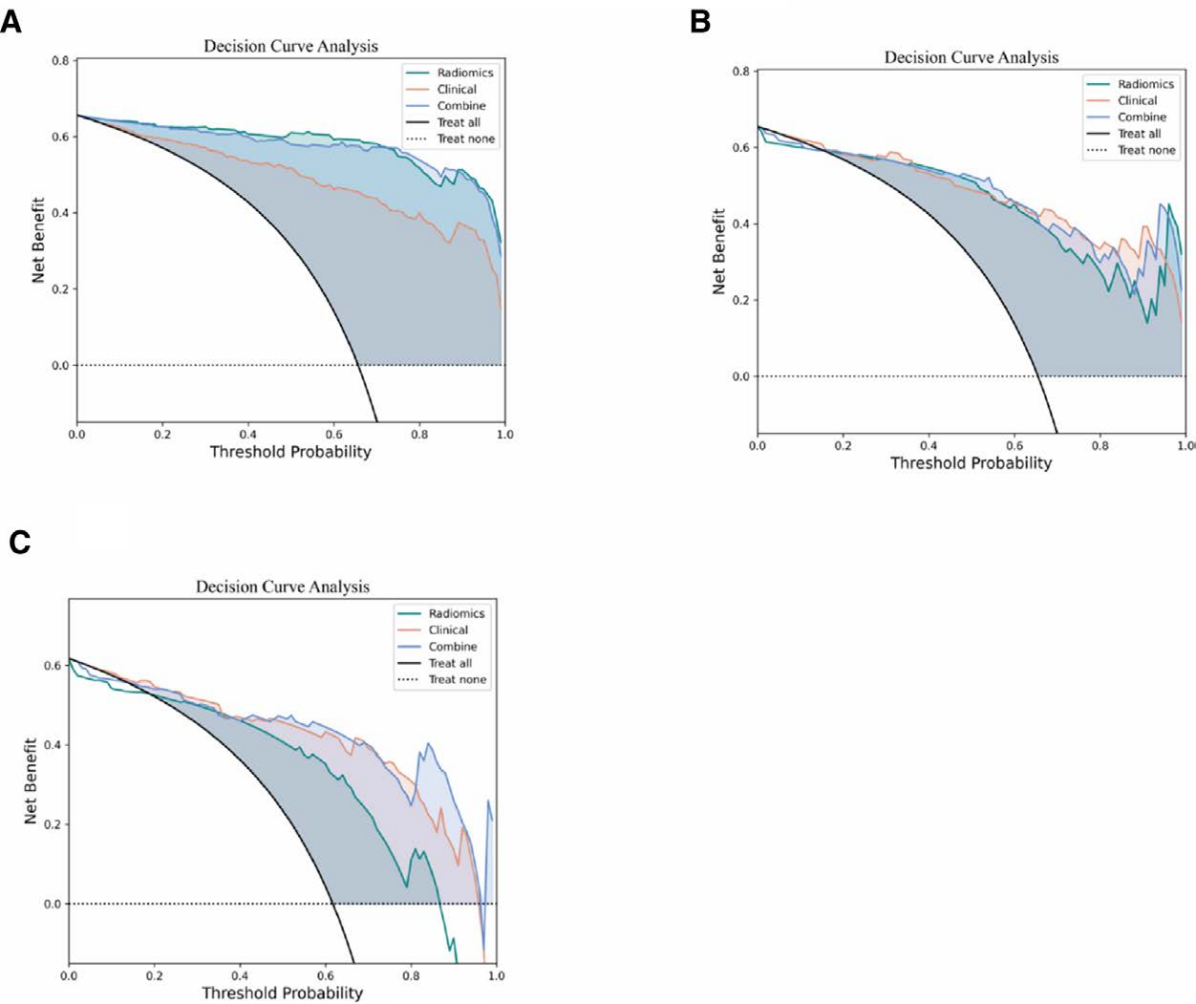
Curve analysis of the 3 models predicting GGNs invasiveness based on the training set (A), testing set (B), and validation set (C). The *x*-axis represents the probability threshold, and the *y*-axis represents the net benefit rate. The dashed line represents the assumption that all GGNs patients have noninvasive lesions, while the solid black line represents the assumption that all GGNs patients have invasive lesions. GGN = ground-glass nodule.

The clinical utility of the radiomics, clinical, and combined models was further evaluated using DCA across the training, testing, and validation cohorts, as presented in Figure 5. In the training set (Fig. 5A), the joint model consistently demonstrated superior net benefit compared to the clinical model, radiomics model, and both “treat all” and “treat none” strategies when the threshold probability ranged from 0.05 to 0.85. In the test set (Fig. 5B), the joint model exhibited optimal net benefit within the threshold probability range of 0.05–0.75. In the validation set (Fig. 5C), the joint model maintained its net benefit advantage across the threshold probability range of 0.05–0.70, demonstrating its robust generalization performance across different scanning devices. Of particular note, the net benefit advantage of the combined model over the single model was most pronounced and consistent across all 3 datasets.

Comparative results of the 3 models in the validation set (Delong test, net reclassification index [NRI], and integrated discrimination improvement [IDI]) are detailed in Table 5. The Delong test, NRI, and IDI were used to compare the different predictive models. The NRI_*P* between the clinical model and the combined model was 0.035, indicating a significant difference, while the IDI between the radiomics model and the combined model was 0.006, suggesting that the combined model outperformed both the standalone clinical and radiomics models.

**Table 5:** Results of the Delong test analysis for the radiomics model, clinical model, and combined model.

Models	Validation cohort				
	Delong_*P*	NRI	NRI_*P*	IDI	IDI_*P*
Clinical model vs radiomics model	0.792	0.083	0.079	−0.005	0.519
Clinical model vs combine model	0.968	0.107	0.035	0.001	0.506
Radiomics model vs Combine model	0.514	0.024	0.262	0.006	0.497

NRI: Represents the increase in the proportion of correctly reclassified samples. If NRI > 0, it indicates a positive improvement, meaning that the new model’s predictive ability has improved compared to the old model. If NRI < 0, it indicates a negative improvement, meaning that the new model’s predictive ability has declined. If NRI = 0, it is considered that the new model has not improved. IDI: The larger the IDI, the better the predictive performance of the new model compared to the old model. If IDI > 0, it indicates a positive improvement, meaning that the new model’s predictive ability has improved compared to the old model. If IDI < 0, it indicates a negative improvement, meaning that the new model’s predictive ability has declined. If IDI = 0, it is considered that the new model has not improved. A *P*-value < .05 indicates that there is a significant difference.

IDI = integrated discrimination improvement, NRI = net reclassification index.

## 4. Discussion

Previous studies show that lung cancer has the highest mortality rate among malignant tumors.^[7]^ About 2.2 million new cases and 1.8 million deaths because of lung cancer were reported globally in 2020, which accounted for 21% of cancer-related mortality and posed a major challenge to human health.^[8]^ Early diagnosis and clinical intervention are particularly important for patient prognosis.^[9]^ Radiomics is an emerging field of quantitative imaging in recent years, at the intersection of computerized machine learning and radiology^[10,11]^. Computers can extract phenotypic features that are normally unrecognizable for visual assessment, resulting in a high-dimensional data space suitable for machine learning. The computer also provides a noninvasive method for improving disease detection and diagnosis, treatment planning, and follow-up by extracting high-throughput imaging data features.^[12-15]^

LR, RF, and SVM are common algorithms in machine learning.^[16,17]^ LR is an algorithm used for handling binary or multi-class classification problems.^[18]^ It uses a logistic function (sigmoid function) to convert the output of a linear regression model into a probability value between 0 and 1. RF is a type of decision tree algorithm, which is an ensemble learning method that constructs multiple decision trees to improve classification or regression accuracy.^[19]^ SVM is a supervised learning algorithm primarily used for data classification, capable of classifying high-dimensional data into a small number of categories, thereby achieving category distinction within a short time.^[20]^ Although the performance of LR, RF, and SVM was statistically equivalent in our test cohort (Table S1, Supplemental Digital Content), we ultimately selected the LR-based combined model for final recommendation. This decision was driven by several practical and clinical considerations crucial for potential translation into practice. First, LR provides inherent interpretability through feature coefficients and odds ratios, allowing clinicians to understand the weight and direction of influence for each predictor (e.g., air bronchogram, specific radiomics features).^[21]^ This transparency is a significant advantage over more complex “black-box” ensembles like RF or kernel-based methods like SVM.^[22]^ Second, given our sample size, the simpler LR model presents a lower risk of overfitting to spurious patterns and demonstrated excellent calibration (Fig. 4). Finally, the use of LR aligns with common practice in clinical prediction modeling, enhancing the comparability of our findings with existing literature and facilitating future test efforts.

Numerous studies have reported the values of GGNs clinical characteristics, imaging morphological features, radiomics features, and various machine learning models in predicting GGNs invasiveness.^[13,23-25]^ Hui et al conducted a retrospective study of 382 cases of solitary pGGNs confirmed as adenocarcinoma by pathology.^[11]^ They established a combined model with 3 clinical radiological features (including age, gender, and mean CT value) and radiomics features, with AUCs of 0.856, 0.859, and 0.765 in the training, testing, and validation cohorts, respectively. The combined model significantly outperformed the clinical model and radiomics model. In this study, univariate analysis showed no significant difference in age and gender between the invasive and noninvasive groups. Sun et al retrospectively analyzed 395 cases of pGGNs, and combined radiomics features and imaging features, including size, edge, and spiculation, to build a combined prediction model, which also achieved good prediction in invasiveness (AAH/AIS and MIA/IAC).^[26]^ The models established by selecting different clinical or imaging parameters and radiomics features have shown strong predictive ability in GGNs invasiveness, but a consensus model has not yet been established.

In the multivariate LR analysis of this study, “mean length” and “air bronchogram” were identified as independent predictive factors. The predictive ability of “Mean length” may encompass or represent some of the local feature information such as lobulation and Burr. From 2 different and complementary dimensions – the overall morphology of GGN and its internal fine structure – it provides the most core discriminative information. The diagnostic threshold for the mean length of GGN was 0.85 cm. A meta-analysis included 16 studies and 2564 pGGNs (761 IAs and 1803 non-IAs) and concluded that the cutoff value of mean diameter is 10.4 mm for the diagnosis of invasive lung adenocarcinoma with high diagnostic efficiency.^[27]^ One study showed that a pGGN with a diameter of 10 mm had a specificity of 100% for diagnosing the invasive lung adenocarcinoma, though there are fewer reports on the diagnostic performance of the mean length.^[28,29]^ Additionally, 217 (60.3%) GGN cases showed an air bronchogram in the study,^[11,30]^ with 50 (23%) cases in the noninvasive group and 167 (77%) cases in the invasive group. That is similar to the results reported by Zhang et al, who found that 210 cases exhibited an air bronchogram, with 16.6% of the noninvasive lesions and 82.3% of the invasive lesions.^[31]^ The models, particularly the clinical-imaging combined model, show good diagnostic performance for predicting invasive lung adenocarcinoma in patients with GGNs.

In many studies, the mean CT value has been shown to be important for diagnosing GGNs invasiveness.^[4,32,33]^ However, this study reported that the mean CT value only showed statistical significance in the training set of the chi-square test, had no statistical significance in the testing set, validation set, and multivariate LR. Few studies have also reported that the mean CT value of pGGNs was not related to the invasiveness of lung adenocarcinoma,^[34]^ but it may be related to the size, density, and delineation method of the cases.

This study has the following limitations. First, the model constructed in this study is mainly based on data from a single center, and its performance has not yet been verified in independent, multi-center external datasets. This may limit its wide clinical application. Second, the relatively small sample size may limit the statistical power of the analysis and increase the risk of overfitting, especially in multivariate and machine learning models (such as LASSO). Moreover, all the enrolled patients in this study received surgical treatment, so the retrospective nature of the study may lead to selection bias. The conclusions of the study may mainly be applicable to the patient group with surgical resectability, and caution should be exercised when extending to a broader group of advanced patients. To enhance the generalizability and reliability of the results, future studies should include larger-scale, multi-center cohorts and adopt a prospective design, and the population should be diverse. In the future, traditional radiomics and deep learning models will also be systematically compared, which will be a very crucial and interesting extension direction in future work.

## 5. Conclusion

In conclusion, the radiomics features extracted from high-resolution computed tomography images have value in predicting invasive lung adenocarcinoma in GGNs. The combination of radiomics features and clinical features is better than the use of radiomics features alone, which may be beneficial for the preoperative evaluation and prognosis of patients.

## Author contributions

**Conceptualization:** Changjun Zheng.

**Data curation:** Wei Zhou, Yinshuo Mei, Yang Li.

**Formal analysis:** Wei Zhou, Jing Zhu, Yinshuo Mei, Yang Li.

**Funding acquisition:** Wei Zhou.

**Investigation:** Jing Zhu.

**Methodology:** Yang Li, Ruonan Sun, Changjun Zheng.

**Project administration:** Ruonan Sun, Changjun Zheng.

**Supervision:** Ruonan Sun, Changjun Zheng.

**Writing – original draft:** Wei Zhou.

## Supplementary Material


