# Supplementary material for: Predictive value of high-resolution computed tomography radiomics for assessing the invasiveness of pulmonary adenocarcinoma presenting as ground-glass nodules: A retrospective study
Source: Medicine (Baltimore). 2026 Mar 6;105(10):e47964. doi: 10.1097/MD.0000000000047964 (PMC12975227; doi:10.1097/MD.0000000000047964)
Supplement: Supplementary file 1 [file medi-105-e47964-s001.docx]

**Table S1.** Pairwise comparison of classifier performance (DeLong's test) for the model on the test set.

|  | Combine  LR Model | Combine  RF Model | Combine  SVM Model | Radiomics  LR Model | Radiomics  RF Model | Radiomics  SVM Model | Clinical  LR Model | Clinical  RF Model | Clinical  SVM Model |
| --- | --- | --- | --- | --- | --- | --- | --- | --- | --- |
| Combine  LR Model | - | 0.353 | 0.802 | 0.514 | 0.267 | 0.100 | 0.968 | 0.383 | 0.756 |
| Combine  RF Model | 0.353 | - | 0.382 | 0.603 | 0.719 | 0.739 | 0.293 | 0.718 | 0.480 |
| Combine  SVM Model | 0.802 | 0.382 | - | 0.623 | 0.260 | 0.116 | 0.968 | 0.400 | 0.820 |
| Radiomics  LR Model | 0.514 | 0.603 | 0.623 | - | 0.344 | 0.049 | 0.792 | 0.576 | 0.974 |
| Radiomics  RF Model | 0.267 | 0.719 | 0.260 | 0.344 | - | 0.970 | 0.360 | 0.912 | 0.485 |
| Radiomics  SVM Model | 0.100 | 0.739 | 0.116 | 0.049 | 0.970 | - | 0.364 | 0.934 | 0.487 |
| Clinical  LR Model | 0.968 | 0.293 | 0.968 | 0.792 | 0.360 | 0.364 | - | 0.153 | 0.393 |
| Clinical  RF Model | 0.383 | 0.718 | 0.400 | 0.576 | 0.912 | 0.934 | 0.153 | - | 0.273 |
| Clinical  SVM Model | 0.756 | 0.480 | 0.820 | 0.974 | 0.485 | 0.487 | 0.393 | 0.273 | - |

**Notes：**Pairwise comparison of classifier performance using DeLong’s test was conducted on the test set across nine models: combined (LR, RF, SVM), radiomics-based (LR, RF, SVM), and clinical-only (LR, RF, SVM) models.

**Table S2.** Using the variance inflation factor (VIF) to test the results of the clinical characteristics that were significant at P < 0.05 from the single-factor analysis

| **variable name** | **VIF value** |
| --- | --- |
| Mean length(mm) | 1.7135 |
| Pleura Involve | 1.4868 |
| Burr | 1.4682 |
| Nodule classification | 1.3817 |
| lobulation | 1.3772 |
| air bronchogram | 1.1469 |
| Vacuole | 1.0441 |

**Notes:** Generally speaking, when VIF is less than 5 (the strict standard), it indicates that there is no significant multicollinearity issue among the variables.


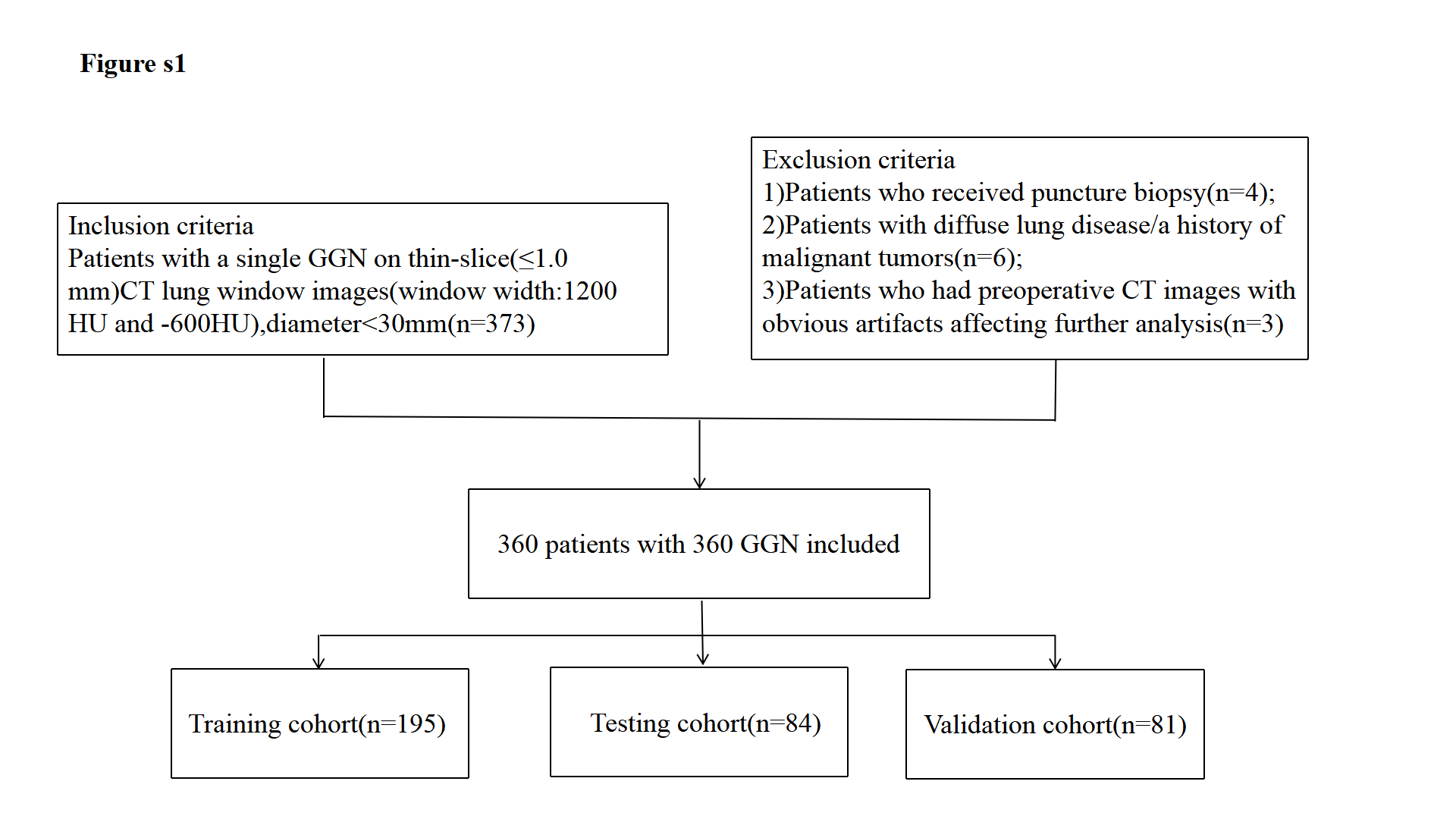


**Figure S1.** The flowchart of study population.
